# Radiation Risk Perception, Breast Cancer Anxiety, and Mental Distress among Women Who Participated in Breast Cancer Screening Programs in Minamisoma City: Five Years after Fukushima’s 2011 Triple Disaster

**DOI:** 10.31662/jmaj.2025-0472

**Published:** 2026-06-05

**Authors:** Ryo Takahashi, Mika Nashimoto, Akihiko Ozaki, Michio Murakami, Yudai Kaneda, Chika Yamamoto, Naomi Ito, Hiroaki Saito, Toyoaki Sawano, Arinobu Hori, Ayu Ajitomi, Kenji Gonda, Masahiro Wada, Kazunoshin Tachibana, Tohru Ohtake, Masaharu Tsubokura, Hiromichi Ohira

**Affiliations:** 1 Department of Clinical Oncology, Akita Red Cross Hospital, Akita, Japan; 2 Kameda Medical Center Breast Center, Chiba, Japan; 3 Teikyo University Graduate School of Public Health, Tokyo, Japan; 4 Breast and Thyroid Center, Jyoban Hospital of Tokiwa Foundation, Fukushima, Japan; 5 Research Center for Community Health, Minamisoma Municipal General Hospital, Minamisoma, Japan; 6 Department of Thyroid and Endocrinology, Fukushima Medical University, Fukushima, Japan; 7 Clinical Training Center, Jyoban Hospital of Tokiwa Foundation, Iwaki, Japan; 8 Department of Health Risk Communication, Fukushima Medical University School of Medicine, Fukushima, Japan; 9 Center for Infectious Disease Education and Research, The University of Osaka, Suita, Japan (current address); 10 Department of Radiation Health Management, Fukushima Medical University School of Medicine, Fukushima, Japan; 11 Department of Internal Medicine, Soma Central Hospital, Soma, Japan; 12 Department of Surgery, Jyoban Hospital of Tokiwa Foundation, Iwaki, Japan; 13 Hori Mental Clinic, Minamisoma, Japan; 14 Department of Breast Surgery, Fukushima Medical University, Fukushima, Japan; 15 Department of Breast Surgery, Utsunomiya Central Clinic, Utsunomiya, Japan; 16 Department of Surgery, Minamisoma Municipal General Hospital, Minamisoma, Japan

**Keywords:** breast neoplasms, mass screening, radiation exposure, mental health, disasters

## Abstract

**Introduction:**

The 2011 Fukushima nuclear disaster following the Great East Japan Earthquake did not cause significant physical health effects, but long-term mental distress persists, partly driven by radiation risk perception. Women, particularly those undergoing breast cancer screening in affected areas, may experience heightened breast cancer anxiety and mental distress. The interrelationships among radiation risk perception, breast cancer anxiety, and mental distress remain unclear. This study aimed to elucidate these associations among women in Minamisoma, one of the municipalities most severely affected by the disaster.

**Methods:**

A self-administered questionnaire survey was conducted among 479 women who attended breast cancer screening at two core breast cancer care centers located more than 20 km north of the Fukushima Daiichi Nuclear Power Plant. This cross-sectional study assessed breast cancer anxiety, risk perception of breast cancer and radiation exposure, and mental distress. Mental distress was evaluated using the Kessler Psychological Distress Scale (K6) scale, with scores ≥5 considered significant. Descriptive analyses and regression models were constructed to explore the relationships among these three outcomes.

**Results:**

Among participants, 47.4% (227/479) reported moderate mental distress. Higher odds of general breast cancer anxiety were observed among those who perceived a high likelihood of radiation-induced breast cancer (odds ratio [OR], 2.99; 95% confidence interval [CI]: 1.76-5.08; p < 0.001). Higher odds of mental distress were also associated with belief in radiation-induced breast cancer (OR, 1.76; 95% CI: 1.19-2.59; p = 0.004). However, breast cancer anxiety itself was not significantly associated with mental distress (OR, 1.29; 95% CI: 0.83-2.01).

**Conclusions:**

Approximately half of the women experienced moderate mental distress five years after the disaster. The results suggest that mental distress is heightened by radiation risk perception and underlying general health concerns, rather than specific factors such as breast cancer anxiety.

## Introduction

On March 11, 2011, the Great East Japan Earthquake triggered a tsunami and nuclear accident ^(1), (2)^. Studies have indicated that radiation exposure levels in Fukushima Prefecture following the disaster were not high enough to cause a significant increase in cancer rates or cases of acute radiation syndrome ^(3)^. Nevertheless, various health problems occurred in the local population ^(4), (5), (6), (7), (8), (9)^. One of the most serious of these is psychological problems ^(10), (11)^. In fact, a high percentage of evacuees in Fukushima Prefecture experienced mental distress and traumatic reactions ^(12)^. In addition, mental health problems are known to have been aggravated in past radiation disasters, such as the Chernobyl Nuclear Power Plant accident, and mental health effects are a typical health effect of radiation disasters ^(13)^.

A typical factor that worsens mental health after a radiation disaster is the risk perception of radiation exposure ^(11)^. That is, residents with high radiation risk perception tend to selectively pay attention to rumors and other anxiety-provoking elements in the local news and, as a result, express a strong sense of anxiety about radiation-induced disorders and illnesses ^(14)^. A 20-year post-Chernobyl nuclear accident review revealed that clean-up workers at the plant experienced rates of depression, anxiety, and medically unexplained physical symptoms two to four times higher than control groups ^(13)^. The severity of these conditions was strongly correlated with the workers’ perception of risk ^(13)^. Furthermore, a study of evacuees in Fukushima Prefecture found similar results, with a positive correlation between risk perception and mental distress ^(11)^.

Of note, even after international organizations such as the United Nations Scientific Committee on the Effects of Atomic Radiation concluded that radiation-related adverse health effects following the Fukushima nuclear accident were negligible, mental health issues associated with radiation risk perception have persisted ^(3)^. For instance, a study conducted four years post-disaster in Kawauchi Village found that approximately half of the residents believed radiation exposure would cause health problems ^(3)^. Moreover, research on residents in evacuation zones seven years after the disaster revealed that while mental distress scores decreased over time, they remained elevated ^(14)^. These findings underscore the importance of recognizing that radiation risk perception may have long-term mental health impacts, independent of scientific conclusions about actual radiation risks.

In this context, we focused on women undergoing breast cancer screening. Breast cancer is the most common cancer worldwide ^(15)^, and ionizing radiation is a known risk factor for the disease ^(16)^. Moreover, women, who are at higher risk of developing breast cancer, tend to have higher radiation risk perception than men ^(17), (18)^. Therefore, women living in radiation-affected areas may experience heightened anxiety about breast cancer and an increased psychological burden. Importantly, breast cancer has an established screening program, and biennial mammography has been shown to reduce mortality by 10%-20% ^(19)^. For women with high radiation risk perception, breast cancer screening sessions could provide valuable opportunities for information sharing and dialogue about radiation risks and breast cancer concerns. This approach may help alleviate anxieties and promote informed decision-making about health management in post-disaster contexts.

Based on these backgrounds, we formulated and tested three hypotheses in this study: 1) Women attending breast cancer screening in areas affected by a radiation disaster may have higher levels of mental distress compared with the general population; 2) Higher perceived radiation risk is associated with increased anxiety about breast cancer, and 3) Higher perceived radiation risk is associated with increased mental distress, with breast cancer anxiety potentially acting as a mediating factor.

This study targeted women undergoing breast cancer screening in areas affected by the Great East Japan Earthquake and the Fukushima Daiichi nuclear disaster. The objectives were: (1) to assess the prevalence of mental distress, and (2) to clarify the interrelationships among radiation risk perception, breast cancer-related anxiety, and overall mental distress. Through this study, we sought to refine the understanding of the psychological impacts of radiation-related concerns in an unprecedented post-disaster context and to deepen knowledge of the long-term mental health implications for communities affected by nuclear disasters.

## Materials and Methods

### Setting and participants

**Figure 1.**
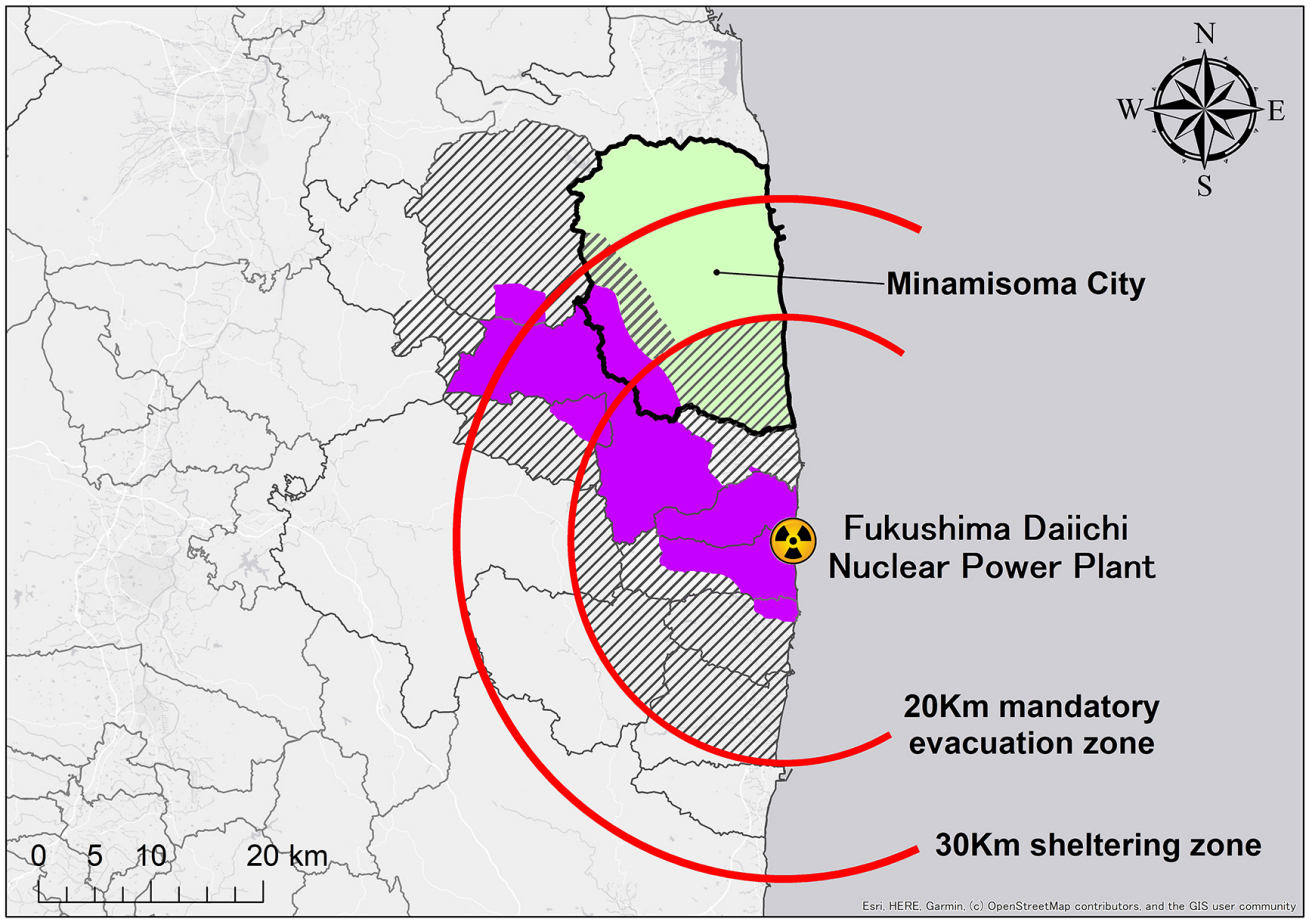
Geographical Context: Minamisoma City and the FDNPP. Minamisoma City (in light green) is situated 14-38 km north of the FDNPP. Following the March 11, 2011, earthquake and tsunami, a hydrogen explosion occurred at FDNPP’s reactor 1 on March 12, 2011. Consequently, areas within a 20 km radius were designated as mandatory evacuation zones, while those within a 30 km radius became sheltering zones. The evacuation zone was expanded on April 22, 2011 (indicated by diagonal stripes and purple). Subsequent modifications lifted evacuation orders in certain areas (also shown with diagonal stripes) as restoration progressed. However, as of November 30, 2023, evacuation orders remained in effect for most of the area colored in purple, where severe contamination persisted near the power plant. Minamisoma Municipal General Hospital and Watanabe Hospital, located 23 km and 25 km north of the FDNPP, respectively, are both situated within Minamisoma City. This image, reproduced from our previous open-access study under the CC BY-NC license and used with permission from Environmental Systems Research Institute (ESRI) Japan Corporation, illustrates the evolving evacuation zones over time in relation to Minamisoma City and the FDNPP ^(32), (33)^. FDNPP: Fukushima Daiichi Nuclear Power Plant.

This cross-sectional study was conducted at Minamisoma Municipal General Hospital (MMGH), located 23 km north of the Fukushima Daiichi Nuclear Power Plant, and Watanabe Hospital (WH), located 25 km north of the nuclear power plant (Figure 1). MMGH and WH suspended outpatient services immediately after the earthquake but resumed them in June 2011. In August 2011, a breast cancer specialist at WH moved to MMGH to resume breast cancer treatment for residents, and since then, MMGH has been the only medical institution in the Soso area, the northern coastal area of Fukushima Prefecture, with a breast cancer specialist ^(7), (20)^. Therefore, these two hospitals are considered the central medical institutions for breast cancer treatment in the Soso region.

A total of 611 women (428 [70.0%] at MMGH and 183 [30.0%] at WH) who visited outpatient clinics at MMGH and WH were included in the survey. The survey was conducted at MMGH from October 17, 2016, to March 28, 2017, and at WH from December 2, 2016, to February 27, 2017. The authors prepared questionnaires with partially different content for women with symptoms and women who had no symptoms and were seen for screening purposes, asking about symptoms in the former group and what form of screening was used in the latter. Of these, only women who had no symptoms and who were examined for breast cancer screening were included in this analysis. In addition, we first excluded patients who did not adequately answer the outcome questions of the study, such as “How concerned are you about getting breast cancer in the future?” and “What is the likelihood that you will get breast cancer in later years due to your current radiation exposure?” Ultimately, 479 (78.4% of 611) patients (315 [65.8% of 479] MMGH and 164 [33.4% of 479] WH) were included in the analysis.

### Analytical data

The variables used in this study were as follows.

#### Sociodemographic factors

Respondents were asked about their age, education, employment, marital and childbearing history, family composition before and after the disaster, and whether their current address was the same as before the disaster.

#### General health factors

General health factors included self-rated health, current medications, smoking history, and alcohol consumption.

#### Factors related to exposure to information on health and illness

Respondents were asked about their level of interest in information on health, frequency of exposure to information on health, frequency of Internet use, and sources of information they trust. Respondents were asked about the following trusted sources of information: family and friends; television; radio; newspapers; magazines; national government; local government; email newsletters; personal websites/blogs; company or medical institution websites; and social networking services.

#### Basic factors related to breast cancer screening

Basic factors related to breast cancer screening included the type of breast cancer screening, history of breast cancer screening, time since the last screening, and whether abnormalities were detected at the last screening.

#### Clinical factors related to breast cancer

Respondents were asked about clinical factors related to breast cancer, such as whether they had a family history of cancer and whether they performed breast self-examination.

#### Factors related to breast cancer-related knowledge and risk perception

We developed 10 breast cancer knowledge items based on prior studies and assessed perceived risk, including the importance of screening, future anxiety about breast cancer and other cancers, and perceived likelihood of developing breast cancer later due to current radiation exposure.

#### Factors related to patients’ mental distress

We also assessed mental distress using the Kessler Psychological Distress Scale (K6), a screener for depression and anxiety, classifying participants as ≥5 (moderate distress) or ≤4 (no moderate distress).

### Data analysis

We conducted five analyses. First, the proportion of participants with K6 scores ≥5, as well as health-related factors, including self-rated health, smoking status, and alcohol consumption, were compared with national estimates from the 2016 Comprehensive Survey of Living Conditions ^(21)^ after age standardization using the 2015 Japanese standard population ^(22)^.

After age standardization, the prevalence of mental distress among participants in the present study was 44.9% (95% confidence interval [CI]: 38.5%-51.3%), whereas the corresponding proportion in the national survey was 31.8%. In the present study, the age-standardized prevalence of poor current self-perceived health status (defined as responses of “Not very healthy” or “Unhealthy”), current smoking, and current alcohol drinking were 35.7% (95% CI: 31.5%-39.9%), 21.7% (95% CI: 19.0%-24.5%), and 47.9% (95% CI: 43.6%-52.2%), respectively. In contrast, the corresponding national estimates were 14.6%, 9.8%, and 29.1%, respectively, and all of these indicators were higher in the study population than in the national data (Supplementary Table 1).

Second, descriptive analyses were conducted for all variables, including sociodemographic, health, and other information factors, as well as factors related to breast cancer and mental distress among participants. Third, to identify factors associated with risk perception of radiation exposure, a multivariable binomial logistic regression model was created for the variable “How likely do you think breast cancer is to occur in later years under your current radiation exposure status?” The outcome was divided into two categories: “very likely, rather likely,” and “rather unlikely, very unlikely.” Multivariable analysis was performed using the backward stepwise variable selection method (inclusion criteria, p < 0.1). The variance inflation factor (VIF) was estimated using the Stata command “collin” for the variables used in the final model to assess multicollinearity. Fourth, to identify factors associated with anxiety about getting breast cancer, a multivariable binomial logistic regression model was created for the variable “How anxious are you about getting breast cancer in the future?” In this case, the responses “very anxious and rather anxious” were grouped as “anxious” as the primary outcome. Multivariable analysis and VIF estimation were conducted in the same way. In univariate analysis, “anxiety about cancers other than breast cancer” was associated but was not included in the multivariable model. Finally, K6-defined mental distress was modeled using multivariable binomial logistic regression. Analysis and VIF checks followed the same procedures, and we also fit a model forcing inclusion of breast cancer anxiety if stepwise selection excluded it. VIF values were low across all final models. Analyses were performed using Stata/IC version 15.0.

Artificial Intelligence (AI)-assisted tools were used only for proofreading and limited text revision, specifically Claude 3.5 (Anthropic) and ChatGPT 5 (OpenAI). All final decisions regarding content and analysis were made by the human authors, and the AI tools served to supplement, but not replace, human expertise and judgment.

### Ethics approval

This study was approved by the MMGH Ethics Committee (30-10) and Fukushima Medical University (3064). Trained staff obtained written informed consent from screening participants after a brief explanation. Data were anonymized, consent could be withdrawn before submission, privacy was strictly protected, and no financial incentives were provided.

## Results

Table 1 presents demographic and health-related characteristics of 479 survey participants. Municipality check-ups were most common (60.4%, 282/467), with 87.8% (418/476) having previous check-ups and 79.1% (329/416) visiting within two years of their last check-up.

**Table 1.** Demographic and Health-Related Characteristics of Survey Participants.

Variable	Total (N = 479)
**Hospital where the examination was received (n, %)**	
Minamisoma Municipal General Hospital	315 (65.8)
Watanabe Hospital	164 (34.2)
**Type of breast cancer screening (n, %)**	
Municipality health check-up	282 (60.4)
Private health check-up	172 (36.8)
Direct outpatient visit	11 (2.4)
Workplace health check-up	2 (0.4)
Missing	12
**History of breast cancer screening (n, %)**	
Yes	418 (87.8)
No	58 (12.2)
Missing	3
**How many years since the last screening (n, %)**	
Within two years	329 (79.1)
Three years or more	87 (20.9)
Missing	63
**Age (median, range)**	56 (29-85)
**Female (n, %)**	479 (100)
**Family history of cancer (n, %)**	
Yes	385 (80.4)
No	94 (19.6)
**Current self-perceived health status (n, %)**	
Very healthy	17 (3.6)
Relatively healthy	337 (70.9)
Not very healthy	113 (23.8)
Unhealthy	8 (1.7)
Missing	4
**Current diseases under treatment or with aftereffects (n, %, multiple choices allowed)**	
Hypertension	118 (24.6)
Hyperlipidemia (lipid abnormality)	73 (15.2)
Eye diseases	39 (8.1)
Musculoskeletal diseases (osteoporosis, arthritis, etc.)	39 (8.1)
Gastrointestinal, liver, or gallbladder diseases	20 (4.2)
Diabetes	17 (3.6)
Respiratory diseases (pneumonia, bronchitis, etc.)	16 (3.3)
Heart diseases	15 (3.1)
Common cold	14 (2.9)
Injuries (falls, fractures, etc.)	10 (2.1)
Other diseases*	72 (15.0)
None^†^	230 (48.0)
**Smoking history (n, %)**	
Current smoker	54 (11.4)
Former smoker	80 (17.0)
Never smoked	338 (71.6)
Missing	7
**Drinking history (n, %)**	
Current drinker	205 (42.8)
Former drinker	94 (19.6)
Never drank	152 (31.7)
Missing	28
**Educational attainment (n, %)**	
Junior high school	31 (6.6)
High school	247 (52.2)
Vocational school/Junior college	137 (28.9)
University	58 (12.2)
Missing	6
**Employment status (n, %)**	
Full-time employee	194 (41.0)
Part-time	96 (20.3)
Self-employed	53 (11.2)
Not employed	130 (27.5)
Missing	6
**Marital status (n, %)**	
Married	352 (74.0)
Widowed/divorced	89 (18.7)
Never married	33 (6.9)
Other	2 (0.4)
Missing	3
**Childbirth history (n, %)**	
Yes	417 (87.4)
No	60 (12.6)
Missing	2
**Number of residential relocations following the disaster (n, %)**	
Once	100 (21.1)
Twice	98 (20.7)
Three times	65 (13.7)
Four times	25 (5.3)
Five times or more	18 (3.8)
Zero	168 (35.4)
Missing	5
**Changes in family composition (all types) (n, %)**	
Living with family before and after the disaster	423 (88.3)
Living with family before, but not after the disaster	26 (5.4)
Not living with family before, but living with family after the disaster	6 (1.3)
Not living with family before or after the disaster	24 (5.0)
**Changes in family composition (spouse) (n %)**	
Living with a spouse before and after the disaster	310 (64.7)
Living with a spouse before, but not after the disaster	45 (9.4)
Not living with a spouse before, but living with a spouse after the disaster	13 (2.7)
Not living with a spouse before or after the disaster	111 (23.2)
**Changes in family composition (children) (n %)**	
Living with children before and after the disaster	217 (45.3)
Living with children before, but not after the disaster	82 (17.1)
Not living with children before, but living with children after the disaster	25 (5.2)
Not living with children before or after the disaster	155 (32.4)
**Changes in family composition (other than spouse and children) (n, %)**	
Living together before and after the disaster	178 (37.2)
Living together before, but not after the disaster	69 (14.4)
Not living together before, but living together after the disaster	24 (5.0)
Not living together before or after the disaster	208 (43.4)
**Frequency of exposure to health-related information (n, %)**	
Every day	85 (18.3)
Every few days	142 (30.5)
Once a week	96 (20.7)
Once a month	124 (26.7)
Never	18 (3.9)
Missing	14
**Trusted sources of information (n, %, multiple choices allowed)**	
Television	385 (80.4)
Family and friends	293 (61.2)
Newspapers	289 (60.3)
Magazines	157 (32.8)
Local government	134 (28.0)
Radio	71 (14.8)
Corporate or medical institution websites	67 (14.0)
National government	60 (12.5)
Social media	15 (3.1)
Personal websites, blogs	13 (2.7)
Email newsletters	5 (1.0)
**Interest in health and illness (n, %)**	
Very interested	189 (39.5)
Somewhat interested	272 (56.8)
Not very interested	18 (3.8)
**Frequency of internet use (n, %)**	
Every day	204 (43.4)
Every few days	53 (11.3)
Once a week	20 (4.3)
Once a month	26 (5.5)
Do not use	167 (35.5)
Missing	9
**K6 score**	
No mild mood disorder (4 points or less)	252 (52.6)
Mild mood disorder present (5 points or more)	227 (47.4)

K6: Kessler Psychological Distress Scale. *Sum of the number of respondents who reported having “other diseases” in the questionnaire and those who reported diseases with a frequency of nine or fewer. ^†^Number of respondents who did not select any disease.

The median age was 56 years (range, 29-85). A total of 80.4% (385/479) had a family history of cancer, and 74.5% (354/475) considered themselves healthy. Hypertension was the most common medical condition (24.6%, 118/479), whereas 48.0% (230/479) had no ongoing treatment. A total of 74.0% (352/476) had a spouse, and 6.7% (32/479) experienced family composition changes after the earthquake.

Regarding health information, 96.1% (447/465) accessed it at least monthly, primarily through television (80.4%, 385/479), family and friends (61.2%, 293/479), and newspapers (60.3%, 289/479). A total of 96.2% (461/479) were interested in health information. Daily Internet use was reported by 43.4% (204/470), whereas 35.5% (167/470) did not use the Internet at all. In terms of mental distress, 47.4% of participants (227/479) had K6 scores of 5 or higher.

Table 2 summarizes breast cancer awareness and risk perceptions. Only 16.5% (77/466) performed monthly self-examinations as recommended. Although 88.9% (426/479) agreed on the importance of breast cancer screening and 77.0% (369/479) agreed on early detection, more than half expressed concerns about various aspects, including discomfort (58.2%, 279/479), embarrassment (60.7%, 291/479), time constraints (62.8%, 301/479), access to facilities (63.9%, 306/479), complex methods (64.9%, 311/479), and high costs (73.9%, 354/479).

**Table 2.** Breast Cancer Awareness, Screening Attitudes, Anxiety, and Radiation Risk Perception.

Variable	Total (N = 479)
**Frequency of breast self-examination (n %)**	
Once a month (regularly)	77 (16.5)
Irregular or never	389 (83.5)
Missing	13
**Thoughts on breast cancer screening (n, %, multiple choices allowed)**	
It is important	426 (88.9)
Early detection is important	369 (77.0)
It is not uncomfortable	200 (41.8)
It is not embarrassing	188 (39.3)
I was able to make time for it	178 (37.2)
Medical facilities are easily accessible	173 (36.1)
The method is easy to understand	168 (35.1)
The cost is low	125 (26.1)
**Knew risk factors for breast cancer (n, %, multiple choices allowed)**	
Family history	385 (80.4)
Smoking	152 (31.7)
Obesity	101 (21.1)
Radiation exposure	98 (20.5)
Aging	72 (15.0)
Alcohol consumption	52 (10.9)
Oral contraceptives	42 (8.8)
**Knew symptoms of breast cancer (n %, multiple choices allowed)**	
Breast lump	445 (92.9)
Dimpling or retraction	293 (61.2)
Nipple discharge (blood)	258 (53.9)
Breast pain	228 (47.6)
Skin redness	74 (15.5)
**Knew facts about mammography (n, %, multiple choices allowed)**	
It is an X-ray imaging device	460 (96.2)
It can be painful	450 (94.1)
It can diagnose before symptoms appear	408 (85.9)
It leads to radiation exposure	375 (78.8)
It is recommended for those 40 years and older	371 (77.9)
It is recommended once every 2 years	335 (70.1)
Benefits outweigh drawbacks (for those 40 and older)	250 (52.3)
It is more effective for those 50 and older	174 (36.6)
**Perception regarding the importance of receiving breast cancer screening (n, %)**	
Very important	330 (68.9)
Somewhat important	146 (30.5)
Not very important	3 (0.6)
Not important at all	0 (0.0)
**Breast cancer anxiety (n, %)**	
Very anxious	100 (20.9)
Somewhat anxious	295 (61.6)
Not very anxious	81 (16.9)
Not anxious at all	3 (0.6)
**Other cancer anxiety (n, %)**	
Very anxious	145 (30.5)
Somewhat anxious	282 (59.2)
Not very anxious	45 (9.5)
Not anxious at all	4 (0.8)
Missing	3
**Radiation risk perception (n, %)**	
Very high	39 (8.1)
Somewhat high	202 (42.2)
Somewhat low	200 (41.8)
Very low	38 (7.9)

Family history was the best-known risk factor (80.4%, 385/479), and breast lumps were the most recognized symptom (92.9%, 445/479).

Almost all participants (99.4%, 476/479) believed in the importance of breast cancer screening. A total of 82.5% (395/479) worried about future breast cancer, with even more participants (89.7%, 427/476) concerned about other cancers. Notably, 50.3% (241/479) believed they might develop cancer due to current radiation exposure.

Table 3 presents a multivariable binomial logistic regression model for the belief that current radiation exposure causes future breast cancer. Significantly higher odds ratios (ORs) were found for those aware of radiation exposure (OR, 2.05; 95% CI: 1.26-3.34; p = 0.004) and obesity (OR, 2.55; 95% CI: 1.55-4.20; p < 0.001) as risk factors. Lower ORs were observed for those able to make time for screening (OR, 0.62; 95% CI: 0.41-0.92; p = 0.019) and those knowledgeable about breast cancer symptoms such as nipple bleeding (OR, 0.63; 95% CI: 0.41-0.96; p = 0.033) and indentation or retraction (OR, 0.52; 95% CI: 0.34-0.81; p = 0.004). Regarding information sources, trusting family and friends increased odds (OR, 1.83; 95% CI: 1.23-2.74; p = 0.003), whereas trusting the national government decreased odds, although not significantly (OR, 0.58; 95% CI: 0.31-1.08; p = 0.088).

**Table 3.** Multivariable Binary Logistic Regression Model for the Radiation Risk Perception (N = 476).

Variable	VIF	OR (95% CI)	p-Value
**Could make time for screening**	1.02		
No		1	
Yes		0.62 (0.41-0.92)	0.019
**Knew radiation exposure as a risk factor for breast cancer**	1.04		
No		1	
Yes		2.05 (1.26-3.34)	0.004
**Knew obesity as a risk factor for breast cancer**	1.04		
No		1	
Yes		2.55 (1.55-4.20)	<0.001
**Knew nipple discharge as a symptom of breast cancer**	1.25		
No		1	
Yes		0.63 (0.41-0.96)	0.033
**Knew dimpling or retraction as a symptom of breast cancer**	1.26		
No		1	
Yes		0.52 (0.34-0.81)	0.004
**Knew that mammography leads to radiation exposure**	1.07		
No		1	
Yes		0.64 (0.40-1.05)	0.076
**Trust family and friends as an information source**	1.01		
No		1	
Yes		1.83 (1.23-2.74)	0.003
**Trust the national government as an information source**	1.05		
No		1	
Yes		0.58 (0.31-1.08)	0.088
**History of diabetes**	1.04		
No		1	
Yes		3.23 (0.95-11.0)	0.061
**History of kidney/prostate disease**	1.05		
No		1	
Yes		7.28 (0.82-64.9)	0.075

CI: confidence interval; OR: odds ratio; VIF: variance inflation factor.

Table 4 presents a multivariable binomial logistic regression model for future breast cancer anxiety. Higher odds of anxiety were found among those believing in a high likelihood of radiation-induced breast cancer (OR, 2.99; 95% CI: 1.76-5.08; p < 0.001) and those interested in health information (OR, 5.17; 95% CI: 1.89-14.2; p = 0.001). Conversely, lower odds were observed for respondents trusting company and medical institution websites (OR, 0.52; 95% CI: 0.27-0.98; p = 0.043) and those considering themselves healthy (OR, 0.48; 95% CI: 0.25-0.94; p = 0.032).

**Table 4.** Multivariable Binary Logistic Regression Model for Breast Cancer Anxiety (N = 475).

Variable	VIF	OR (95% CI)	p-Value
**Radiation risk perception**	1.01		
Considers the possibility low		1	
Considers the possibility high		2.99 (1.76-5.08)	<0.001
**Trust corporate/medical institution websites as an information source**	1.01		
No		1	
Yes		0.52 (0.27-0.98)	0.043
**Interested in health information**	1.00		
No		1	
Yes		5.17 (1.89-14.2)	0.001
**Consider oneself currently healthy**	1.01		
No		1	
Yes		0.48 (0.25-0.94)	0.032

The variable “anxiety about developing cancers other than breast cancer” was excluded from the initial set of covariates when performing the calculations and constructing the final model. CI: confidence interval; OR: odds ratio; VIF: variance inflation factor.

Table 5 presents a multivariable binomial logistic regression model for mental distress using the K6 scale. Higher odds of mental distress were associated with belief in radiation-induced breast cancer (OR, 1.76; 95% CI: 1.19-2.59; p = 0.004), post-earthquake relocation (OR, 1.57; 95% CI: 1.05-2.34; p = 0.028), and previous breast cancer screening (OR, 1.92; 95% CI: 1.04-3.54; p = 0.036). Lower odds were found among those considering themselves healthy (OR, 0.60; 95% CI: 0.39-0.94, *p* = 0.024). Notably, breast cancer anxiety was not significantly associated with mental distress in either the final (Model 1) or forced-entry (Model 2) models.

**Table 5.** Multivariable Binary Logistic Regression Model for Mental Distress Using the K6 Scale.

	Model 1 (N = 464)		Model 2 (N = 464)	
Variable	OR (95% CI)	*p*-value	OR (95% CI)	*p*-value
**Knew dimpling or retraction as a symptom of breast cancer**				
No	1		1	
Yes	0.68 (0.46-1.02)	0.063	0.66 (0.44-0.98)	0.042
**Knew breast cancer screening is recommended from age 40**				
No	1		1	
Yes	0.63 (0.40-1.01)	0.055	0.62 (0.39-0.99)	0.045
**Consider oneself currently healthy**				
No	1		1	
Yes	0.60 (0.39-0.94)	0.024	0.59 (0.38-0.91)	0.018
**Have received breast cancer screening in the past at least once**				
No	1		1	
Yes	1.92 (1.04-3.54)	0.036	1.89 (1.03-3.49)	0.041
**Any relocation following the disaster**				
No	1		1	
Yes	1.57 (1.05-2.34)	0.028	1.54 (1.03-2.30)	0.034
**Radiation risk perception**				
Considers the possibility low	1		1	
Considers the possibility high	1.76 (1.19-2.59)	0.004	1.85 (1.25-2.74)	0.002
**Trust the national government as an information source**				
No	1			
Yes	0.60 (0.33-1.10)	0.098		
**Breast cancer anxiety**				
No			1	
Yes			0.81 (0.49-1.36)	0.432

OR, odds ratio; CI, confidence interval; K6, Kessler Psychological Distress Scale Variation inflation factors for model 1 range from 1.01 to 1.06 and those for model 2 ranged from 1.01 to 1.09. In Model 2, the variable “anxiety about developing breast cancer in the future” was forcibly entered, and the final model was constructed.

## Discussion

This study included women undergoing breast cancer screening at medical institutions located 20-30 km from the Fukushima Daiichi Nuclear Power Plant five years after the disaster. We hypothesized that: 1) Women attending breast cancer screening in areas affected by a radiation disaster may have higher levels of mental distress compared with the general population; 2) Higher perceived radiation risk is associated with increased anxiety about breast cancer, and 3) Higher perceived radiation risk is associated with increased mental distress, with breast cancer anxiety potentially acting as a mediating factor. Results showed that about half of the participants experienced mental distress. Women who believed they were at risk of radiation-induced breast cancer exhibited both breast cancer anxiety and mental distress. However, breast cancer anxiety itself was not associated with mental distress. Thus, hypotheses 1 and 2 were supported, whereas hypothesis 3 was only partially supported, as the mediating effect of breast cancer anxiety on the association between perceived radiation risk and mental distress was not observed.

### Interpretations of the study findings

The prevalence of mental distress among women in this study exceeded national averages. Furthermore, our findings surpassed the level observed in the entire Fukushima population, as evidenced by a previous study examining 100 randomly selected respondents from each municipality in Fukushima Prefecture five years post-disaster, which showed that 29.2% scored 5 or higher on the K6 scale ^(23)^. In the study population, the prevalence of poor self-rated health, current smoking, and current alcohol drinking was higher than that in the national reference data. These differences suggest that our sample is inherently distinct from the national population. Beyond this, interpretation of our findings requires consideration of the disaster-specific context. Indeed, the elevated distress observed in Minamisoma City can be attributed to two major factors. First, the city’s unique geographical position, located 14-38 km from the nuclear power plant, resulted in partial evacuation, with the southern third designated as an evacuation zone. This created a complex situation in which residents outside the evacuation zone faced the difficult decision of whether to evacuate voluntarily. This pressure was particularly acute for women, who often balanced significant responsibilities in both their professional lives and families ^(24), (25)^. Second, despite scientific evidence indicating acceptable levels of radiation exposure in the area ^(26), (27)^, Minamisoma residents were continually exposed to radiation-related misinformation and disinformation ^(28)^. This persistent exposure to conflicting information likely contributed to heightened risk perception regarding radiation-induced health effects, including breast cancer.

In this study, perceived radiation risk was associated with both increased anxiety about developing breast cancer and higher levels of overall mental distress, whereas anxiety about developing breast cancer itself was not significantly associated with mental distress. One possible explanation for this finding is that greater knowledge about breast cancer, including awareness of screening and early symptoms, may enable individuals to perceive breast cancer as a treatable disease, thereby limiting the progression of breast cancer-related anxiety into broader mental distress. In general, higher health literacy has been associated with lower risk perception ^(29)^. Furthermore, undergoing breast cancer screening and resolving uncertainty about one’s health status may help prevent breast cancer-related anxiety from becoming more generalized ^(30)^. Therefore, mental health support in disaster-affected areas should address not only disease-specific anxiety but also generalized health anxiety related to radiation exposure.

### Clinical implications of the study findings

To translate the findings of this study into clinical practice in the context of disaster settings, we propose two key strategies. First, implement brief anxiety risk screening during breast cancer screenings using a short questionnaire that can be completed while patients wait. In parallel, establish standardized protocols enabling staff to identify high-risk individuals and facilitate smooth referrals to mental health services. Second, utilize breast cancer screening as a hub for community education by organizing regular educational sessions on radiation risks and breast cancer, separate from screening appointments, and by establishing clear referral pathways to mental health professionals for patients experiencing significant distress. Notably, a behavioral activation program developed for mothers living in Fukushima demonstrated positive effects on psychological distress and physical symptoms ^(31)^, suggesting the potential benefit of combining follow-up consultations with such interventions for high-risk individuals. Together, these measures would allow early identification and response to mental health concerns without adding undue burden to the screening process, while providing appropriate support and resources to those in need.

### Limitations

This study has several limitations. First, although the two facilities surveyed in Minamisoma City serve a substantial proportion of local breast cancer screening patients, the study was geographically limited and did not include an internal control group because of the study design. To partially address this and provide context for the study population, we compared the prevalence of mental distress and selected basic health indicators―self-rated health, smoking status, and alcohol consumption―with nationally representative data from the Comprehensive Survey of Living Conditions. Differences between these datasets complicate the interpretation of our findings and limit their generalizability to other settings.

Second, the single post-earthquake, cross-sectional survey design precludes assessment of changes in the immediate aftermath or earlier periods, limiting temporal insights and inherently restricting the ability to infer causal relationships between variables. Third, the study’s focus on women undergoing breast cancer screening, chosen because of the established link between radiation exposure and breast cancer and the existence of evidence-based screening programs, may not reflect the experiences of the general population or those with other health concerns.

Despite these limitations, this study provides valuable insights into radiation risk perception in a post-disaster context, particularly in relation to breast cancer screening. It should also be noted that these facilities have extensive experience and established expertise in conducting questionnaire surveys targeting individuals attending screening ^(32), (33)^. Future longitudinal studies with broader geographical and population scope could address these limitations and further enhance understanding of the long-term psychological impacts of radiation disasters on health-seeking behaviors and risk perceptions.

### Conclusions

This study found that women undergoing breast cancer screening in post-disaster Fukushima experienced higher levels of mental distress compared with the national average. Radiation risk perception was strongly associated with both breast cancer anxiety and overall mental distress. Notably, while radiation risk perception was linked to mental distress, it was not associated with breast cancer-specific anxiety. This suggests that, in this context, general health concerns may have a greater impact on mental health than cancer-specific worries. These findings provide important guidance for shaping breast cancer screening practices in post-disaster settings.

## Article Information

### Acknowledgments

We express our sincere gratitude to the staff of Watanabe Hospital and Minamisoma Municipal Hospital for their invaluable assistance in data collection and management. We also thank Masatsugu Tanaki at MMGH for creating the map used in this study. Ryo Takahashi and Mika Nashimoto equally contributed to the work.

### Author Contributions

Conception and design of the study: Ozaki and Murakami. Provision of study materials or patients and collection and assembly of data: Ozaki, Sawano, and Ohira. Writing this paper: Takahashi, Nashimoto, Ozaki, and Murakami. Data analysis, interpretation, and critical revision of the paper: All authors. All the authors read the final draft and approved the submission.

### Conflicts of Interest

Dr. Ozaki received personal fees from MNES Inc., Kyowa Kirin Co., Ltd., Becton, Dickinson and Company, Pfizer Inc., Daiichi Sankyo Inc., and Taiho Pharmaceutical Co., Ltd., outside the scope of the submitted work.

### Approval Code Issued by the Institutional Review Board (IRB) and the Name of the Institution that Granted the Approval

MMGH Ethics Committee (Approval No. 30-10).

Fukushima Medical University (Approval No. 3064).

## Supplement

Supplementary Material

## References

[1] Brumfiel G, Cyranoski D. Quake sparks nuclear crisis. Nature. 2011;471(7338):273-5. 21412302 10.1038/471273a

[2] Shibahara S. The 2011 Tohoku earthquake and devastating tsunami. Tohoku J Exp Med. 2011;223(4):305-7. 21478655 10.1620/tjem.223.305

[3] Orita M, Hayashida N, Nakayama Y, et al. Bipolarization of risk perception about the health effects of radiation in residents after the accident at Fukushima nuclear power plant. PLoS One. 2015;10(6):e0129227. 26057539 10.1371/journal.pone.0129227 PMC4461282

[4] Moriyama N, Morita T, Nishikawa Y, et al. Association of living in evacuation areas with long-term care need after the Fukushima accident. J Am Med Dir Assoc. 2022;23(1):111-6.e1. 34146522 10.1016/j.jamda.2021.05.030

[5] Nomura S, Gilmour S, Tsubokura M, et al. Mortality risk amongst nursing home residents evacuated after the Fukushima nuclear accident: a retrospective cohort study. PLoS One. 2013;8(3):e60192. 23555921 10.1371/journal.pone.0060192 PMC3608616

[6] Ohira T, Nakano H, Okazaki K, et al. Trends in lifestyle-related diseases and their risk factors after the Fukushima Daiichi nuclear power plant accident: results of the comprehensive health check in the Fukushima health management survey. J Epidemiol. 2022;32 Suppl XII:S36-46. 36464299 10.2188/jea.JE20210386 PMC9703921

[7] Ozaki A, Nomura S, Leppold C, et al. Breast cancer patient delay in Fukushima, Japan following the 2011 triple disaster: a long-term retrospective study. BMC Cancer. 2017;17(1):423. 28629330 10.1186/s12885-017-3412-4 PMC5477136

[8] Ozaki A, Saito H, Kaneda Y, et al. Long-term uptake rate of a breast cancer screening program in Fukushima, Japan, following the 2011 Triple Disaster: a retrospective observational study. Sci Rep. 2023;13(1):6654. 37095136 10.1038/s41598-023-33717-8 PMC10123585

[9] Yamashita S, Suzuki S, Suzuki S, et al. Lessons from Fukushima: latest findings of thyroid cancer after the Fukushima nuclear power plant accident. Thyroid. 2018;28(1):11-22. 28954584 10.1089/thy.2017.0283 PMC5770131

[10] Shigemura J, Terayama T, Kurosawa M, et al. Mental health consequences for survivors of the 2011 Fukushima nuclear disaster: a systematic review. Part 1: psychological consequences. CNS Spectr. 2021;26(1):14-29. 32192553 10.1017/S1092852920000164

[11] Suzuki Y, Yabe H, Yasumura S, et al. Psychological distress and the perception of radiation risks: the Fukushima health management survey. Bull World Health Organ. 2015;93(9):598-605. 26478623 10.2471/BLT.14.146498 PMC4581639

[12] Yabe H, Suzuki Y, Mashiko H, et al. Psychological distress after the Great East Japan Earthquake and Fukushima Daiichi Nuclear Power Plant accident: results of a mental health and lifestyle survey through the Fukushima Health Management Survey in FY2011 and FY2012. Fukushima J Med Sci. 2014;60(1):57-67. 25030715 10.5387/fms.2014-1

[13] Bromet EJ, Havenaar JM. Psychological and perceived health effects of the Chernobyl disaster: a 20-year review. Health Phys. 2007;93(5):516-21. 18049228 10.1097/01.HP.0000279635.14108.02

[14] Kashiwazaki Y, Takebayashi Y, Murakami M. Relationships between radiation risk perception and health anxiety, and contribution of mindfulness to alleviating psychological distress after the Fukushima accident: cross-sectional study using a path model. PLoS One. 2020;15(7):e0235517. 32628692 10.1371/journal.pone.0235517 PMC7337340

[15] Arnold M, Morgan E, Rumgay H, et al. Current and future burden of breast cancer: global statistics for 2020 and 2040. Breast. 2022;66:15-23. 36084384 10.1016/j.breast.2022.08.010 PMC9465273

[16] Engel CL, Sharima Rasanayagam M, Gray JM, et al. Work and female breast cancer: the state of the evidence, 2002-2017. New Solut. 2018;28(1):55-78. 29658425 10.1177/1048291118758460

[17] Oishi K, Orita M, Taira Y, et al. Risk perception of health risks associated with radiation exposure among residents of Okuma, Fukushima Prefecture. Int J Environ Res Public Health. 2021;18(24):13208. 34948816 10.3390/ijerph182413208 PMC8703485

[18] Rasmussen J, Ewald J, Sterner T. Gender and life-stage dependent reactions to the risk of radioactive contamination: a survey experiment in Sweden. PLoS One. 2020;15(4):e0232259. 32353020 10.1371/journal.pone.0232259 PMC7192462

[19] Van Ourti T, O’Donnell O, Koç H, et al. Effect of screening mammography on breast cancer mortality: quasi-experimental evidence from rollout of the Dutch population-based program with 17-year follow-up of a cohort. Int J Cancer. 2020;146(8):2201-8. 31330046 10.1002/ijc.32584 PMC7065105

[20] Ozaki A, Nomura S, Leppold C, et al. Breast cancer provider interval length in Fukushima, Japan, after the 2011 triple disaster: a long-term retrospective study. Clin Breast Cancer. 2020;20(2):e127-50. 31980405 10.1016/j.clbc.2019.07.008

[21] Comprehensive survey of living conditions 2016: summary results [Internet]. Ministry of Health, Labour and Welfare. 2025 [cited 2025 Dec 24]. Japanese. Available from: https://www.mhlw.go.jp/toukei/saikin/hw/k-tyosa/k-tyosa16/

[22] Regarding the standard population for age-adjusted mortality rates [Internet]. Ministry of Health, Labour and Welfare. 2020 [cited 2025 Dec 24]. Japanese. Available from: https://www.mhlw.go.jp/toukei/saikin/hw/jinkou/kakutei20/dl/14_nencho.pdf

[23] Fukasawa M, Kawakami N, Umeda M, et al. Environmental radiation level, radiation anxiety, and psychological distress of non-evacuee residents in Fukushima five years after the Great East Japan Earthquake: multilevel analyses. SSM Popul Health. 2017;3:740-8. 29349260 10.1016/j.ssmph.2017.09.002 PMC5769096

[24] Hirohara M, Ozaki A, Tsubokura M. Determinants and supporting factors for rebuilding nursing workforce in a post-disaster setting. BMC Health Serv Res. 2019;19(1):917. 31783755 10.1186/s12913-019-4765-y PMC6884864

[25] Kaneda Y, Ozaki A, Murakami M, et al. Impact of large-scale disasters on breast cancer care: a qualitative analysis of patient experiences during the 2011 triple disaster in Fukushima, Japan. Int J Disaster Risk Reduc. 2024;109:104582.

[26] Tsubokura M, Kato S, Morita T, et al. Assessment of the annual additional effective doses amongst minamisoma children during the second year after the Fukushima Daiichi Nuclear Power Plant disaster. PLoS One. 2015;10(6):e0129114. 26053271 10.1371/journal.pone.0129114 PMC4460119

[27] Tsubokura M, Gilmour S, Takahashi K, et al. Internal radiation exposure after the Fukushima nuclear power plant disaster. JAMA. 2012;308(7):669-70. 22893159 10.1001/jama.2012.9839

[28] Sawano T, Ozaki A, Hori A, et al. Combating ‘fake news’ and social stigma after the Fukushima Daiichi Nuclear Power Plant incident-the importance of accurate longitudinal clinical data. QJM. 2019;112(7):479-81. 30778551 10.1093/qjmed/hcz049

[29] Murakami M, Orita M, Sekiya N. Chapter 14. Radiation risk perception after the Fukushima disaster. In: Kamiya, K, Ohto, H, Maeda, M, editors. Health effects of the Fukushima nuclear disaster. Cambridge (MA): Academic Press; 2022. p. 247-63.

[30] Osterø J, Siersma V, Brodersen J. Breast cancer screening implementation and reassurance. Eur J Public Health. 2014;24(2):258-63. 23788014 10.1093/eurpub/ckt074

[31] Imamura K, Sekiya Y, Asai Y, et al. The effect of a behavioral activation program on improving mental and physical health complaints associated with radiation stress among mothers in Fukushima: a randomized controlled trial. BMC Public Health. 2016;16(1):1144. 27825326 10.1186/s12889-016-3819-x PMC5101788

[32] Kaneda Y, Ozaki A, Murakami M, et al. Health information seeking on the Internet among patients with and without cancer in a region affected by the 2011 Fukushima triple disaster: cross-sectional study. JMIR Cancer. 2024;10:e49897. 39167438 10.2196/49897 PMC11375390

[33] Ozaki A, Murakami M, Nomura S, et al. Overall health information exposure, its barriers and impacts on attitude toward healthcare among cancer patients. The long-term aftermath of the 2011 triple disaster in Fukushima, Japan: a single institution cross-sectional study. Health Inform J. 2021;27(2):1460458221996420. 10.1177/1460458221996420 33878956

