# Supplementary material for: Radiation Risk Perception, Breast Cancer Anxiety, and Mental Distress among Women Who Participated in Breast Cancer Screening Programs in Minamisoma City: Five Years after Fukushima’s 2011 Triple Disaster
Source: JMA J. 2026 Jun 5;9(4):750–63. doi: 10.31662/jmaj.2025-0472 (PMC13453577; doi:10.31662/jmaj.2025-0472)
Supplement: Supplementary file 1 [file 2433-3298-9-4-0750-s001.pdf]

Supplementary Table 1: Age-standardized prevalence of selected health-related indicators compared with national estimates.

Variable	Age-standardized prevalence % (95% CI)	National estimate (%)
**Current self-perceived health status**		
Poor†	35.7 (31.5-39.9)	14.6
**Smoking history**		
Current smoking	21.7 (19.0-24.5)	9.8
**Drinking history**		
Current drinking	47.9 (43.6-52.2)	29.1
**Mental distress**		
K6 score ≥5	44.9 (38.5-51.3)	31.8

†Poor self-perceived health status was defined as responses of “Not very healthy” or “Unhealthy.”

CI=confidence interval
